# *In vitro* and *Ex vivo* study targeting the development of a *Lavandula stoechas* L. (*Ustukhuddūs*) loaded Unani Transdermal patch: Implication of Unani Medicine in the treatment of *Nisyan* (Dementia)

**DOI:** 10.1016/j.heliyon.2024.e25284

**Published:** 2024-01-26

**Authors:** Farhath Fathima A, Imran Khan, Mohammed Irfhan N, Zaheer Ahmed N, Noman Anwar, Mohd Shahnawaz Khan, Dharmendra Kumar Yadav, Shariq Shamsi, Anas Shamsi

**Affiliations:** a Regional Research Institute of Unani Medicine, Chennai, 600013, India; b National Institute of Unani Medicine, Bengaluru, 560091, India; c Government Unani Medical College, Chennai, 600106, India; d Central Council for Research in Unani Medicine, New Delhi, 110025, India; e Department of Biochemistry, College of Science, King Saud University, Riyadh, Kingdom of Saudi Arabia; f Gachon Institute of Pharmaceutical Science and Department of Pharmacy, College of Pharmacy, Gachon University, Incheon, Republic of Korea; g Centre of Medical and Bio-allied Health Sciences Research, Ajman University, United Arab Emirates

**Keywords:** Ustukhuddūs, Unani transdermal patch, Essential oil, Sustained-release, Ex vivo permeation, Skin irritation

## Abstract

*Ustukhuddūs* (*Lavandula stoechas* L.) has been extensively used orally and topically in treating various neurological disorders, including dementia. The optimum potential of traditional dosage forms of *Ustukhuddūs* is limited for various reasons. Transdermal drug delivery system (TDDS) is a novel means of drug delivery and is known to overcome the drawbacks associated with traditional dosage forms. The current study aimed at fabricating and evaluating *Ustukhuddūs* hydro-alcoholic extract (UHAE) and essential oil (UEO) loaded matrix-type transdermal patches having a combination of hydrophilic - hydroxyl propyl methyl cellulose (HPMC) and hydrophobic - ethyl cellulose (EC) polymers. ATR–FTIR, DSC, XRD, and SEM analysis were carried out to study drug–polymer interactions, confirming the formation of developed patches and drug compatibility with excipients. We assessed the fabricated patches to evaluate their physicochemical properties, *in vitro* drug release, and permeation characteristics via *ex vivo* experiments. The physicochemical characteristics of patches showcased the development of good and stable films with clarity, smoothness, homogeneity, optimum flexibility and free from causing skin irritancy or sensitization. *In vitro* drug release and *ex vivo* permeation profile of developed patches were evaluated employing Franz diffusion cells. UHAE and UEO patches exhibited a cumulative drug release of 81.61 and 85.24 %, respectively, in a sustained-release manner and followed non-Fickian release mechanisms. The *ex vivo* permeation data revealed 66.82 % and 76.41 % of drug permeated from UHAE and UEO patches, respectively. The current research suggests that the formulated patches are more suitable for TDDS and hold potential significance in the treatment of dementia, contributing to enhanced patient compliance, thereby highlighting the implication of Unani Medicine in Nisyan (Dementia) treatment.

## 1 Introduction

The term drug delivery system (DDS) encompasses a range of physicochemical technologies designed in a way to regulate the delivery and release of pharmacologically active substances into cells, tissues, and organs ensuring that these active substances can exert their optimal effects. There are various administration modalities, depending on the chosen delivery route; amongst them Transdermal drug delivery system (TDDS) represents an appealing approach attracting researcher's attention across the globe. In recent times, TDSS has garnered significant being an attractive alternative means of drug delivery for oral administration and hypodermic injection owing to the fact that TDDS offers a painless and convenient method, potentially enhancing drug bioavailability, thereby putting minimum burden on the patients [1,2]. Other advantages of TDSS is its ability to circumvent the digestive system, and the potential to evade first-pass metabolism in the liver. Nevertheless, complete potential of TDSS remains untapped due to the inherent barrier presented by the skin. A transdermal patch is a medication-infused patch designed to release drugs into the bloodstream at a controlled rate by permeating through the layers of the skin [3]. Transdermal patches, widely embraced for treating various medical conditions, are adhesive devices that contain drugs with a specific surface area. They provide controlled drug delivery through intact skin, ensuring regulated systemic circulation [4,5]. These patches offer several benefits, including simple preparation, ease of use, high hydrophilic and lipophilic drug capacity, and improved stability for long-term dermal delivery. Another important attribute of a transdermal patch is that it offers controlled and consistent drug delivery, preventing sudden spikes in drug levels in the bloodstream. Thus, this straightforward dosing approach can improve patient adherence to the prescribed drug regimen [4,6]. Additionally, it is very helpful, particularly in patients who receive therapies for longer duration and those who are unable or unwilling to take oral medications [7,8].

Neuropsychiatric conditions, viz. Depression, attention deficit hyperactivity disorder (ADHD), Parkinson's disease (PD), and dementia, require medication for longer durations, which is often associated with dropout, poor patient compliance, and increased burden of care ^[^. For these conditions, long-acting formulations are beneficial as they ensure a sustained level of medication in the bloodstream, thereby consistently providing the required symptom relief rather than experiencing sporadic spikes in drug levels. Studies suggest that patients receiving anti-dementia therapies at adequate doses for longer periods slow cognitive decline, decreasing the need for nursing home admissions and lowering overall healthcare expenditures [9,10]. Owing to the patient noncompliance and adverse effects produced by orally administered cholinesterase inhibitors in dementia cases, TDSSs were developed for various acetylcholinesterase inhibitors, including tacrine, physostigmine, phenserine tartrate. However, the only approved and commercialized transdermal patch of acetylcholinesterase inhibitors is the rivastigmine transdermal patch, which is marketed as Exelon® Patch [9,10]. Rivastigmine patch has been reported for its sustained release characteristics and maintaining plasma concentration [11,12]. It was also found satisfactory regarding ease of use and decreased interference with daily life [13].

*Ustukhuddūs* (*Lavandula stoechas* L.- Lamiaceae) is a highly acclaimed drug in Unani Medicine, used to treat various neurological disorders, including epilepsy, catalepsy, dementia, insanity, mental weakness, and obsessive thoughts apart from other systemic afflictions. It is also known as the “Broom of the brain” (a tonic to the brain that clarifies the intellect) [14,15]. The extracts and essential oils of *Ustukhuddūs* have been reported to possess potent hypnotic [16], nootropic [17], anticonvulsant, sedative and antispasmodic [18], anti-apoptotic [19], antioxidant, and anti-inflammatory properties [20]. Unani Medicine indicated both oral administration and topical application of *Ustukhuddūs* paste on the head to treat dementia and catalepsy [15]. While *Ustukhuddūs* demonstrates superior therapeutic benefits through oral administration, its optimal efficacy is limited by challenges, namely, low bioavailability, bulkiness, and the necessity for frequent dosing. The paste formulation has challenges related to inconsistent dosing, limited drug availability due to incomplete release, and difficulties in ensuring patient compliance [21]. All these challenges create a problem and needs to be tackled. Thus, development of polymer-based topical patches of *Ustukhuddūs* may serve this purpose as it will overcome all these drawbacks that are being aced by dosage forms, thereby highlighting the importance of polymer-based topical patches of *Ustukhuddūs* Thus, this study is a first of its kind that is aimed at developing transdermal patches of *Ustukhuddūs* with controlled release properties using a blend of hydrophilic and hydrophobic polymers, incorporating hydro-alcoholic extract and essential oil of *Ustukhuddūs*. The study also involved evaluating the *in vitro* and *ex vivo* permeation as well as sustained release characteristics of these patches.

## 2 Materials and methods

### 2.1 Materials

Hydroxypropyl methylcellulose was purchased from High Purity Laboratory Chemicals Pvt Ltd. (Mumbai). Ethyl cellulose, dibutyl phthalate, dimethyl sulfoxide, and methanol were purchased from Thomas Baker Chemicals Pvt Ltd. (Ambernath). Ethanol was purchased from Jiangyin Import & Export Co. Ltd. (China). Chloroform was purchased from Nice Chemicals Pvt Ltd. (Kochi). Dichloromethane was purchased from Sisco Research Laboratories. (Maharashtra). Glycerine was procured from SD Fine Chemicals Ltd. located in Mumbai. All chemicals and solvents utilized were of analytical grade.

### 2.2 Methods

#### 2.2.1 Preparation of Ustukhuddūs extract and essential oil

Dried aerial parts of *Ustukhuddūs* (*Lavandula stoechas* L.) were procured from Green Earth Products Pvt Ltd, New Delhi. They were authenticated by the Drug standardisation and Research Unit (DSRU), Regional Research Institute of Unani Medicine (RRIUM), Chennai. The plant specimen was preserved in Drug Museum, National Institute of Unani Medicine (NIUM), Bengaluru, with voucher number 115/1S/Res/2022. Extraction was carried out as per the method mentioned by Mustafa SB, 2019 [21]. For extraction, 20 g of crushed *Ustukhuddūs* was macerated with 50 % water and ethanol (Hydroalcoholic) at a ratio of 1: 20 (wt/vol) using a rotary shaker for a total duration of 72 h. The extract was filtered manually using a muslin cloth. The filtrate obtained was re-filtered through Whatman filter paper. The extraction process was conducted in triplicate, and the resulting filtrates were pooled in a beaker. The combined filtrates were then subjected to evaporation under reduced pressure using a Rotary Evaporator, operating at 90 rpm and maintaining a temperature of 40 °C until complete dryness was achieved. Finally, the remnants were collected and dried under a vacuum oven at 40 °C. The extract was weighed, and the yield percentage was determined in relation to the initial quantity of the raw medicinal material. It was stored in an airtight container free from contamination at −4 °C for further use [22]. Essential oil was extracted through a water steam distillation device (Clevenger-apparatus) following the method described by Al-Mariri, 2013 [23].

#### 2.2.2 Formulation of transdermal patches of Ustukhuddūs

Transdermal Patches were developed according to the method mentioned by Patel N A, 2009 [23]. Two matrix-type transdermal patches were prepared using specified amount of *Ustukhuddūs* Hydro-alcoholic Extract (UHAE) and *Ustukhuddūs* Essential Oil (UEO), with two different polymers, hydrophilic Hydroxyl propyl methyl cellulose K4M [HPMC K4M] (100 mg) and hydrophobic Ethyl cellulose [EC] (25 mg) by solvent casting technique. The polymers were dissolved using a precisely optimized solvent blend of chloroform, methanol, and dichloromethane in a 2:2:1 ratio. The calculated amount of UHAE/UEO was then carefully dispersed into the polymeric solutions, ensuring uniform distribution of the drug. Dibutyl phthalate (DBP) was added at 30 % of the total polymer weight as a plasticizer, and Dimethyl sulfoxide (DMSO) was added at 20 % of the total polymer weight as a permeation enhancer (refer to Table 1). The resulting polymeric drug solution was poured into a lubricated square mould using glycerine and left to dry at room temperature, covered with an inverted funnel to maintain a dust-free environment. After 24 h, the dried patches were cut into 1 cm2 pieces using a knife. These transdermal patches were then carefully wrapped in aluminium foil and stored in a desiccator for future use. Similarly, patches without the drug (blank blended patches) were prepared using the same procedure [24].

**Table 1:** Composition of UHAE, UEO and blank blended patches^a^.

Patch	Amount of drug (mg)	Polymer		Plasticizer	Permeation Enhancer	Solvent (v/v) mL
		HPMC (mg)	EC (mg)	DBP (mg)	DMSO (mg)	Chl:MeOH:DCM
UHAE	54	100	25	37.5	25	2:2:1
UEO	25	100	25	37.5	25	2:2:1
Blank	–	100	25	37.5	25	2:2:1

a HPMC indicates hydroxyl propyl methyl cellulose; EC, ethyl cellulose; DBP, dibutyl phthalate; DMSO, dimethyl sulfoxide; Chl, chloroform; MeOH, Methanol; DCM, Dichloromethane; UHAE, Ustukhuddūs hydro-alcoholic extract; UEO, Ustukhuddūs essential oil.

#### 2.2.3 Drug-polymer interaction and compatibility studies

##### 2.2.3.1 ATR-FTIR study

ATR-FTIR analysis was done on the PerkinElmer Spectrum-1 FTIR spectrometer (model: PerkinElmer Spectrum-1, USA.) to assess drug and polymer compatibility following the method described by Suksaeree J, 2015 [25]. This involved capturing IR transmission spectra and identifying characteristic peaks for HPMC, EC, blank patches, UHAE, and UEO matrix patches. The scanning was done at a resolution of 4 cm^−1^ over a wavenumber region of 500–4000 cm^−1^ using the FTIR spectrometer.

##### 2.2.3.2 Differential scanning calorimetry (DSC) study

The endothermic transitions and ingredient compatibility were analyzed using a DSC instrument (model: Netzsch DSC 204, Germany) according to the method provided by Suksaeree J, 2015 [25]. Briefly, DSC pan was hermetically sealed after transferring a sample and subsequently subjected to analysis using the DSC instrument. The temperature range spanned from 20 to 350 °C, employing a heating rate of 10 °C/min within a liquid nitrogen atmosphere. The obtained DSC thermogram was documented, and analyzed.

##### 2.2.3.3 X- ray diffraction (XRD) study

XRD analysis, performed using the D8 Advance Bruker model, Singapore, assessed the compatibility of blank blended patches, UHAE blended patches, and UEO blended patches as stated by Suksaeree J, 2015 [25]. X-ray source was operated at a voltage of 40 kV and a current of 45 mA, utilizing an angular range of 5–40° (2θ) and a step increment of 0.02° (2θ) per second.

##### 2.2.3.4 Scanning electron microscopy (SEM)

Surface and cross-sectional SEM analysis was done of the samples incubated at 37 °C on a glass slide using FEI Quanta 200, MK II, USA with high vacuum and high voltage of 20-kV condition and using Everhart-Thornley detector [25].

#### 2.2.4 Physicochemical evaluation of formulated patches

##### 2.2.4.1 Physical appearance

The formulated patches were evaluated for uniformity, transparency, clarity, color, absence of air bubbles, flexibility, and smoothness [26]. Any patches with flaws, air entrapment, thickness irregularities, or weight inconsistencies were excluded from further testing.

##### 2.2.4.2 Weight uniformity

Each formulated patch was independently weighed using an analytical balance (Shimadzu AX 200, Kyoto, Japan) to determine weight uniformity. Following that, the weight of each patch was compared to the average weight [26].

##### 2.2.4.3 Thickness

The thickness consistency for all formulated patches was assessed using a vernier caliper (from Germany). We took the measurements at six different locations on each patch, and the mean thickness value was computed [26].

##### 2.2.4.4 Drug content

The drug content estimation of the formulated UHAE and UEO patches was studied according to the method mentioned by Latif MS, 2021 [26] using UV–visible spectrophotometer (Shimadzu 1601, Kyoto, Japan) by measuring absorbance at wavelengths of 273 nm and 278 nm, following appropriate dilutions [26].

##### 2.2.4.5 Folding endurance

The test was performed to assess the impact of the plasticizer on the patch. It entails repeatedly folding a patch at a certain spot until a break or crack appears, demonstrating the patch's folding endurance capacity. All other parameters were followed as described by Latif MS, 2021 [26].

##### 2.2.4.6 Moisture uptake

Moisture uptake of the formulated patches was assessed by following the method stated by Latif MS, 2021 [26]. The percent moisture uptake was calculated by determining the difference between the initial and final weights of the patches. Subsequently, the average percent moisture uptake was calculated.

##### 2.2.4.7 Moisture content

The modified patches' moisture content was evaluated by following the method mentioned by Latif MS, 2021 [26]. The percent moisture content was calculated using the following equation:

(1) % Moisture Loss = (wi − wf)/wi × 100

Where wi is the initial patch weight, and wf is the final patch weight.

##### 2.2.4.8 Surface pH

The surface pH of the formulated patches was determined by following the method explained by Latif MS, 2021, using a pH meter (InoLab®, Xylem Analytics, Germany) [26].

##### 2.2.4.9 Percent elongation at break

The percent elongation was measured by recording the length just before the breakpoint. The percentage elongation was calculated by using the following equation.

(2) % Elongation = (L_1_– L_2_)/L_2_ × 100

Where L_1_ is the final patch length before breaking, and L_2_ is the initial patch length.

##### 2.2.4.10 Flatness

The flatness of the patches was calculated by following the method described by Singh A, 2016 [27]. Flatness was computed using the provided equation:

(3) % Constriction = = (L_1_–L_2_)/L_1_ × 100

Where L_1_ is the initial strip length, and L_2_ is the strip length.

#### 2.2.5 Skin irritation study

The potential of developed patches to cause skin irritation or sensitization was assessed through hypersensitivity tests on rat skin. Healthy Wistar strain male albino rats weighing 200-250 gm were procured from a registered breeder of Bengaluru. All experiments were carried out in the Animal House, National Institute of Unani Medicine, Bengaluru. Ethical clearance was obtained from the Institutional Animal Ethics Committee (IAEC) vide No. IAEC/6/19/IA/07. The Draize patch test was employed for skin irritation [26]. Two groups, each containing six rats, were used to assess the skin irritancy in each formulation.

The hypersensitivity tests of the formulated UHAE, UEO, and blank patches were evaluated by following the method described by Latif MS, 2021 [26]. A visual scoring scale was employed to categorize skin irritation levels: “0″ denoted no irritation, “1″ denoted slight irritation, “2″ denoted well-defined irritation, “3″ denoted moderate irritation, and “4″ denoted scar formation on the skin [26].

#### 2.2.6 *In vitro* drug release study

*In vitro* release studies of UHAE and UEO patches were evaluated by following the method mentioned by Baviskar DT, 2013 using a modified Franz diffusion cell using egg shell membrane. For the determination of the drug content of UHAE and UEO patches, samples were analyzed spectrophotometrically at 273 and 278 nm wavelength, respectively, after suitable dilution with phosphate buffer pH 7.4. Each experiment was performed in triplicate [28].

#### 2.2.7 Kinetics of drug release

The release profiles of all batches were analyzed by fitting them to various mathematical models, including Zero order, First order, Higuchi, Hixson and Crowell model, Korsmeyer-Peppas, Hopfenberg, Weibull, MakoidBanakar, Baker-Lonsdale, and Peppas-Sahlin models. This was done to ascertain the kinetics of drug release [26]. The coefficient of correlation and rate constants were determined using Add In Program DD Solver [29].

#### 2.2.8 *Ex vivo* permeation study

*Ex Vivo* permeation studies of UHAE and UEO patches was evaluated in modified Franz diffusion cell by following the method demonstrated by Baviskar DT, 2013 with pig flank skin as the membrane. The pig flank skin was sourced from a local pork slaughterhouse where the animals were slaughtered for human consumption, and no animals were specifically killed for this experiment. The samples were analyzed spectrophotometrically to determine the drug content of UHAE and UEO patches at wavelengths of 273 nm and 278 nm, respectively, after appropriate dilution with fresh medium. Each experiment was conducted in triplicate. Cumulative amounts of drug permeated were calculated in micrograms per square centimetre (μg/cm^2^) and plotted against time [28].

#### 2.2.9 Skin penetration study

Skin penetration studies of UHAE and UEO patches were determined by following the method demonstrated by Salemo C, 2010. Skin drug penetration, denoting the amount retained within the skin, was calculated as the difference between the total applied dose and the amount permeated, combined with the amount present in the remaining sample over the skin [30].

#### 2.2.10 Statistical analysis

The experiments were conducted in triplicate, and the results were presented as the mean ± standard deviation.

## 3 Results and discussion

### 3.1 Organoleptic properties of *ustukhuddūs* extract, essential oil and developed patches

The yield of UHAE and UEO was found to be 30 % and 0.4 % (g/g) respectively. UHAE was greenish-dark brown, highly sticky, and extremely bitter, with a strong aroma, whereas UEO was found to be dark yellow, intensely bitter, with potent camphor aroma. The physical appearance of blank patches was found to be white. In contrast, UHAE and UEO blended patches were noted to be dark brown and pale yellow, respectively, attributed to their distinctive constituent. Developed transdermal patches with appropriate polymeric combinations and other excipients showcased a good film with clarity, smoothness, and homogeneity and were free from the entrapment of any air bubble.

### 3.2 Drug-polymer interaction and compatibility studies

**Fig. 1:**
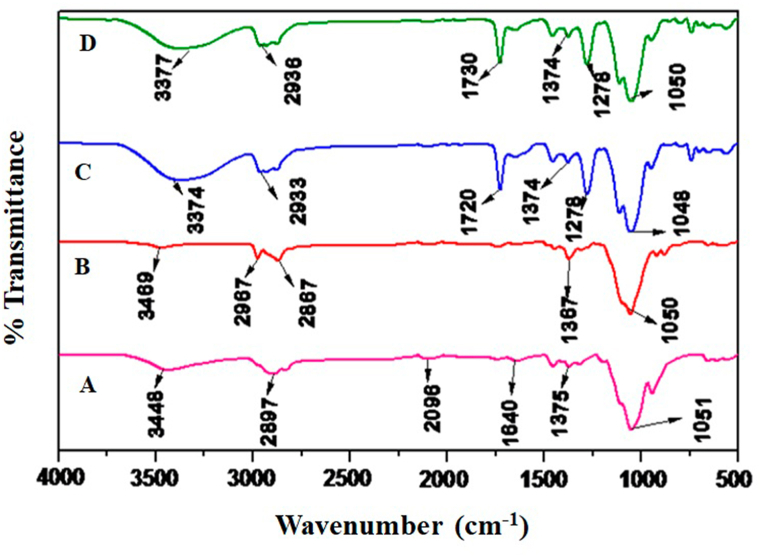
FTIR spectra of (A) HPMC, (B) EC, (C) UHAE patch, (D) UEO patch*. *HPMC indicates hydroxyl propyl methyl cellulose; EC, ethyl cellulose; UHAE, Ustukhuddūs hydro-alcoholic extract; UEO, Ustukhuddūs essential oil.

In the present study, FTIR spectra of HPMC, EC, UHAE, and UEO blended patches were studied and represented in Fig. 1. The FTIR spectrum of HPMC showed a peak at 3448 cm^−1,^ representing –OH stretching vibration. The peaks at 2897 and 2096 cm^−1^ indicate C–H stretching vibration. The peak at 1640 cm^−1^ corresponds to C–O stretching vibration, whereas the peak at 1375 cm^−1^ corresponds to C–O–C stretching, and the peak at 1051 cm^−1^ indicates C–O stretching vibration (Fig. 1A). FT-IR spectrum of EC showed a characteristic peak at 3469 cm^−1^ due to –OH stretching vibration. The C–H stretching vibration peaks were observed at 2967 cm^−1^ and 2867 cm^−1^. The other important peaks at 1367 and 1050 cm^−1^ correspond to C–O–C stretching and C–O stretching vibrations. (Fig. 1B). The FTIR spectrum of the UHAE patch presented characteristic peaks at 3374 cm^−1^ (O–H stretching), 2933 cm^−1^ (C–H stretching), 1720 cm^−1^ (C=O stretching), 1720 cm^−1^ (C=O bending), 1374 cm^−1^ (C–O–C stretching), 1278 cm^−1^ (C–O stretching), 1048 cm^−1^ (C–H bending) (Fig. 1C). Similarly, the FTIR spectrum of UEO patch showed characteristic peaks at 3377 cm^−1^ (O–H stretching), 2936 cm^−1^ (C–H stretching), 1730 cm^−1^ (C=O stretching), 1374 cm^−1^ (C–O–C stretching), 1278 cm^−1^ (C–O stretching) and 1050 cm^−1^ (C–H bending) respectively. (Fig. 1D). The study's findings indicated that the physical mixture displayed noticeable drug peaks alongside the polymer peaks in the spectra. This observation affirms that the drug remained inert with the excipients. All the peaks were found intact, indicating UHAE and UEO's compatibility with other excipients used in the formulation.

**Fig. 2:**
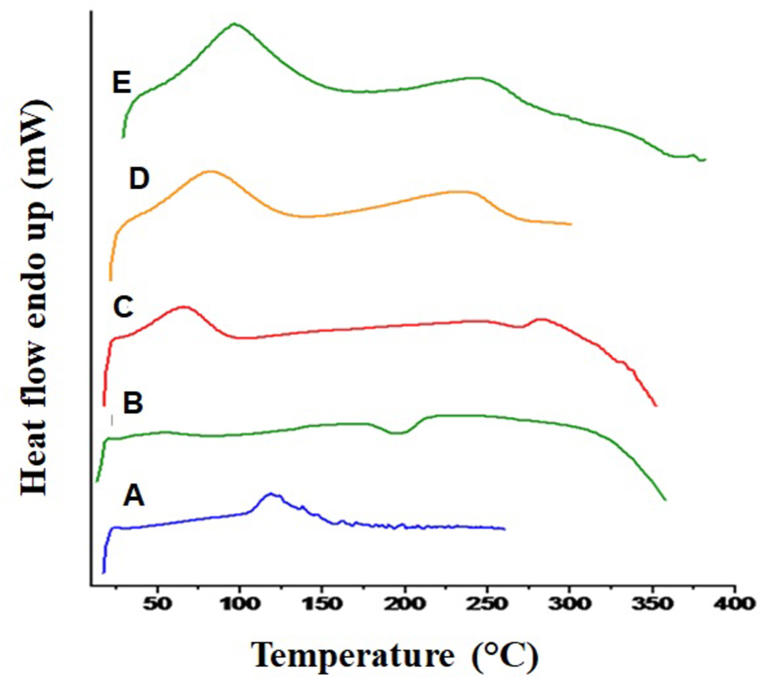
*DSC Thermograms of****(A)****crude hydro-alcoholic extract,****(B)****EC,****(C)****HPMC,****(D)****UEO patch,****(E)****UHAE patch*.

DSC analysis was utilized to investigate the thermal properties of the blended patches containing UHAE and UEO [25]. DSC thermograms of pure UHAE, EC, HPMS, UEO, and UHAE blended patches are shown in Fig. 2. The thermogram of pure UHAE exhibited four endothermic peaks at 118.5 °C, 124.0 °C, 138.1 °C, and 146.6 °C corresponding to its melting point and enthalpy of peak (Δ*H*) was 139.7 J/g (Fig. 2A). The DSC thermogram of EC showed a broad endothermic peak appears at 55.9 °C (ΔH -14.26 J/g) and an exothermic peak at 194.9 °C (Δ*H* -23.2 J/g) (Fig. 2B)**.** The thermograms of HPMC showed a broad endothermic peak at 65.7 °C, and the enthalpy of peak (ΔH) was 124.8 J/g, and exothermic peak at 268.7 °C. The enthalpy of peak (ΔH) was −14.55 J/g, which may be attributed to the dehydration of water molecules (Fig. 2C). The thermogram of the UEO blended patch shows two broad endothermic peaks, the first peak at 82.4 °C (ΔH 235.9 J/g) and the second at 235 °C (ΔH 204.2 J/g) (Fig. 2D). The thermogram of the UHAE blended patch showed a very broad endothermic peak at 95.9 °C (ΔH 379.6 J/g) initially, followed by an exothermic peak at 237.3 °C (ΔH 160.8 J/g) (Fig. 2E).

The present study revealed a disappearance of the endothermic peak of pure UHAE in the developed UHAE patch, which may be attributed to the proper embedment of the extract into HPMC and EC, respectively. The thermogram of the physical mixture closely resembled that of the drug, indicating a high dispersion of the drug within the polymer. Furthermore, it suggested that the drug did not form any complex with the polymer used in the formulation. In the DSC thermograms of UHAE and UEO patches, no new peaks or alterations in peak shape and onset were observed. A minor shift in the HPMC and EC peak in these thermograms could be attributed to moisture in the patch. These findings affirm the maintained chemical integrity of the patch and the absence of interactions between the drug and polymers.

**Fig. 3:**
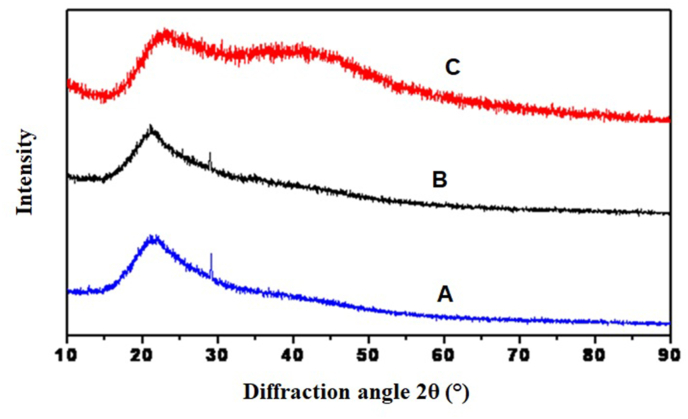
X-ray diffractograms of **(A)** UHAE patch, **(B)** Blank patch, **(C)** UEO patch. UHAE indicates Ustukhuddūs hydro-alcoholic extract; UEO, Ustukhuddūs essential oil.

The XRD pattern of blank, UHAE, and UEO blended patches are depicted in Fig. 3. The XRD technique was utilized to investigate the compatibility of the polymer combination with the drug and identify and characterize the crystalline and amorphous forms of the sample [25]. The XRD patterns of blank patches developed from HPMC and EC showed a peak around 20.92°, indicating semi-crystalline characters, whereas the UHAE and UEO blended patches showed a broad diffraction halo of the amorphous region. The disappearance of HPMC and EC crystalline peaks in UHAE and UEO blended patches might have resulted from high drug dispersion within the polymers, indicating the compatibility of developed patches.

**Fig. 4:**
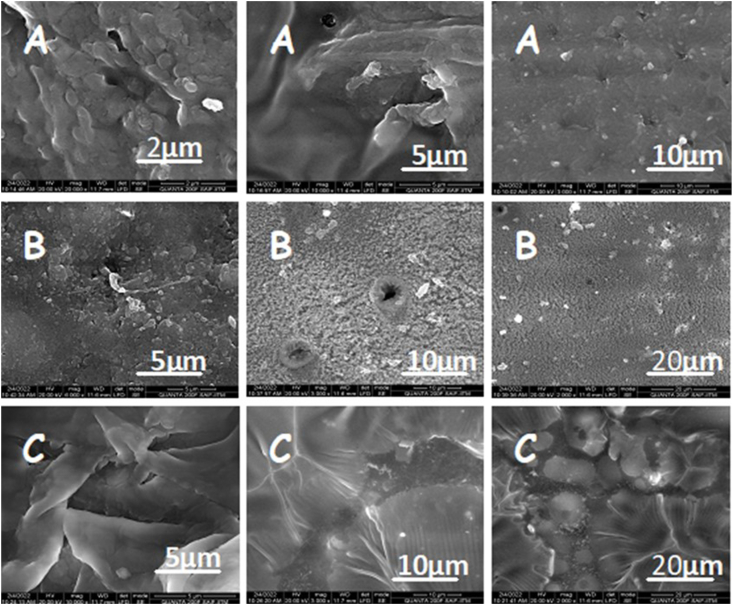
Representative SEM Images of **(A)** Blank patch, **(B)** UHAE patch, **(C)** UEO patch. UHAE indicates Ustukhuddūs hydro-alcoholic extract; UEO, Ustukhuddūs essential oil.

High-resolution images of the surface and cross-section of blank, UHAE, and UEO blended patches were captured using SEM (Fig. 4). SEM analysis deciphered that the surface of blank blended patches displayed a uniformly smooth and dense texture without visible pores. However, upon the incorporation of crude UHAE and UEO into the blank blended patches, the surface of UHAE and UEO blended patches appeared rough and uneven. This was attributed to the dispersed clusters and aggregates resulting from crude UHAE and UEO in the blended patch matrix.

### 3.3 Physicochemical evaluation of formulated patches

The physicochemical characteristics of blank, UHAE, and UEO blended patches are shown in Table 2. The mean weight (in mg) of blank, UHAE, and UEO blended patches was recorded to be 53.51 ± 1.230, 70.256 ± 2.009, and 58.486 ± 0.217, respectively. The mean thickness (in mm) of blank, UHAE, and UEO blended patches was recorded to be 0.31 ± 0.02, 0.436 ± 0.015, and 0.31 ± 0.017, respectively. The weight uniformity and thickness variation between different developed patches was within acceptable range. Low SD values were found in the weight and thickness of patches, indicating uniformity of each patch and reproducibility of the procedure followed for developing patches. The mean percentage of drug content of UHAE and UEO blended patches was found to be 83.94 ± 1.12 and 85.49 ± 0.67 %, respectively. The findings demonstrated that the formulation achieved consistent drug content and minimal variation among the various patches developed, ensuring even drug distribution. Transdermal patches exhibiting higher folding endurance are regarded as superior quality, as they resist breakage or damage more effectively [31]. In the present study, the folding endurance values of blank, UHAE, and UEO blended patches were 42.666 ± 4.041, 26.293 ± 0.577, and 49.333 ± 3.511, respectively. The folding endurance of developed patches was recorded to be satisfactory, indicating optimum flexibility of patches with less brittleness. However, the UHAE blended patch showed comparatively less flexibility, which may be attributed to the high moisture content in the patch.

**Table 2:** Physicochemical properties of Formulated Patches (n = 3).

Parameters	Formulations		
	UHAE patch	UEO Patch	Blank Patch
Weight Uniformity (mg)	70.256 ± 2.009	58.486 ± 0.217	53.51 ± 1.230
Thickness (mm)	0.436 ± 0.015	0.31 ± 0.017	0.31 ± 0.02
Drug Content (%)	83.94 ± 1.12	85.49 ± 0.67	–
Folding Endurance (no's)	26.293 ± 0.577	49.333 ± 3.511	42.666 ± 4.041
Moisture Uptake (%)	2.983 ± 0.120	2.18 ± 0.055	2.383 ± 0.070
Moisture Content (%)	3.186 ± 0.083	2.786 ± 0.070	2.846 ± 0.111
Surface pH	5.726 ± 0.073	5.17 ± 0.088	5.903 ± 0.023
Percentage Elongation (%)	17.666 ± 4.041	37.666 ± 4.041	35.33 ± 4.041
Flatness (%)	100	100	100

All values are expressed as mean ± SD (*n* = 3). UHAE indicates Ustukhuddūs hydro-alcoholic extract; UEO, Ustukhuddūs essential oil.

The percentage of moisture uptake for the blank, UHAE, and UEO blended patches was 2.383 ± 0.070, 2.983 ± 0.120, and 2.18 ± 0.055, respectively. The percentage of moisture content for the blank, UHAE, and UEO blended patches was recorded to be 2.846 ± 0.111, 3.186 ± 0.083, 2.786 ± 0.070, respectively. The percentage moisture uptake and content of developed patches was within the acceptable range. The low moisture uptake could protect the developed patches from microbial contamination and reduce bulkiness. In contrast, the low moisture content of the patches could help the patches remain stable and reduce brittleness [28]. The surface pH values of the blank, UHAE, and UEO blended patches were 5.903 ± 0.023, 5.726 ± 0.073 and 5.17 ± 0.088, respectively. The pH of developed patches was within the acceptable range, indicating the absence of skin irritancy or sensitization [26]. The mean percentage elongation of blank, UHAE, and UEO blended patches was 35.33 ± 4.041, 17.666 ± 4.041, and 37.666 ± 4.041 % respectively. Typically, for transdermal applications, patches need flexibility, allowing them to conform to the skin's movements without breaking. The percentage elongation of blank and UEO blended patches indicated better elasticity than UHAE. The percentage flatness of all the developed patches was 100 %. No variation in the strip lengths before and after a longitudinal cut was observed in the developed patches, indicating 100 % flatness and 0 % constriction. Hence, when applied to the skin, the developed patches maintain a smooth and uniform surface [27].

### 3.4 Skin irritation study

The skin irritation study result revealed that upon application of developed UHAE and UEO patches on healthy male Wistar rats for 24 h, no edema or erythema was observed on the site of application of patches. The result indicates that the developed UHAE and UEO blended patches are non-irritable to the skin and safe for transdermal application.

### 3.5 *In vitro* drug release study

**Fig. 5:**
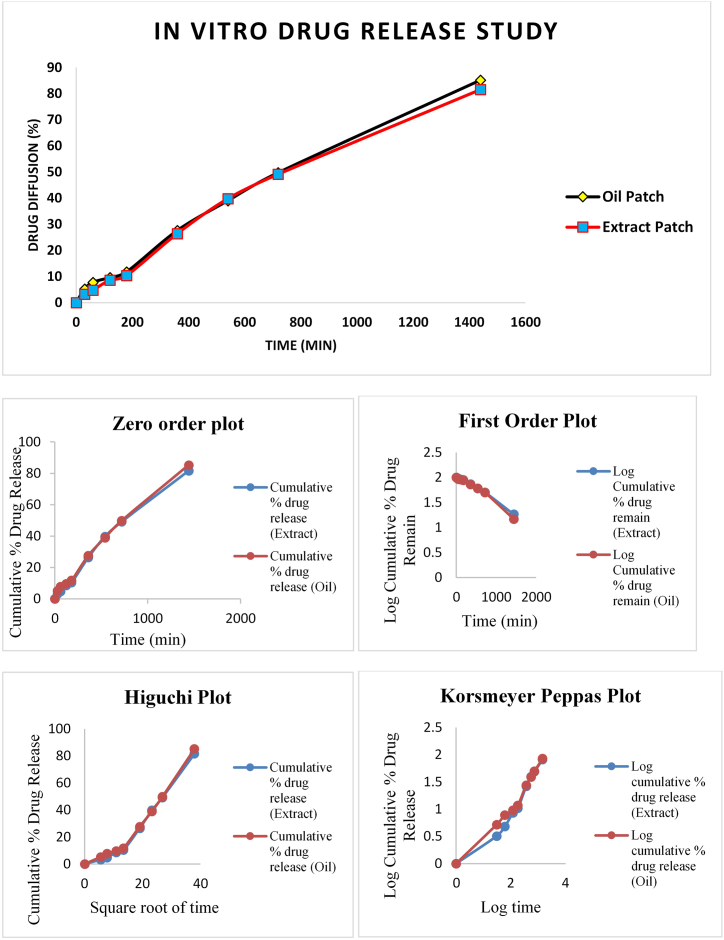
In vitro drug release profile of optimized patches and *in vitro* release kinetics of zero order, first order, Higuchi's and Korsmeyer peppas models. Oil patch indicates Ustukhuddūs essential oil patch; extract patch, Ustukhuddūs hydro-alcoholic extract patch. All values are expressed as mean ± SD (n = 3).

The determination of release rates is a vital tool for evaluating all controlled drug delivery systems. Conducting diffusion studies on patches ensures a constant drug permeation rate. Consistently maintaining a higher drug concentration on the stratum corneum's surface than the drug concentration in the body is vital to achieving the intended controlled drug release. In the present study, evaluation of *in vitro* drug release profile of UHAE and UEO patches containing extract and essential oil in concentrations of 54 mg/cm^2^ and 25 mg/cm^2,^ respectively, was performed on Franz diffusion cells using eggshell membrane, which is known to be similar to human stratum corneum. The results showed a notable initial burst release within the first 7 h, followed by a gradual approach to a plateau. This pattern implies the controlled-release behaviour of the matrix formulations over a 24-h period. The final cumulative percentage drug release from UHAE and UEO patches after 24 h was recorded to be 81.61 ± 0.37 % and 85.24 ± 0.42 % respectively (Fig. 5). The rapid initial burst release observed within the first 7 h is likely attributed to the swift diffusion of the drug from the surface of the patches and into the receptor medium. The developed patches' controlled-release profile is promising, particularly for chronic conditions such as dementia. These patches have the potential to maintain consistent plasma levels, making them valuable as long-acting formulations in treating chronic conditions.

Additionally, based on the results obtained from moisture uptake and porosity studies through SEM, it can be inferred that HPMC polymers likely created space and a significant free volume within the drug-blended patches. This phenomenon could have increased molecular mobility and segmental relaxation, consequently enhancing drug diffusion. It is evidenced that the matrix-type patches had an amorphous region, leading to enhanced drug diffusion [32]. Both the UHAE and UEO patches released the drug in a sustained manner. However, the percentage cumulative release of the developed UHAE patch was found to be significantly lower than that of the developed UEO patch. The enhanced drug release profile of the UEO patch may well be understood as essential oils, and their volatile constituents are known to be good penetration enhancers [33].

### 3.6 Drug release kinetics

Different mathematical models used to identify drug release patterns from UHAE and UEO blended patches are shown in Table 3 & Fig. 5. Based on the higher regression values (r^2^), the best-fit model for both UHAE and UEO blended patches was the Weibull model with R^2^ values of 0.9985 and 0.9967, respectively. Korsmeyer-Peppas model for both the developed patches is between 0.5 and 1, thus indicating non-Fickian release mechanisms, implying that the release of the formulated UHAE and UEO patches was accomplished through a diffusion mechanism coupled with enhanced erosion and swelling.

**Table 3:** Regression parameters of formulated patches after fitting the drug release data to various release kinetic models.

Mathematical models	Extract Patch	Essential oil Patch
Zero- order Model R^2^ k_0_ (h^−1^)	0.9826 0.061	0.9893 0.063
First – Order Model R^2^ k_1_ (h^−1^)	0.986 0.001	0.9790 0.001
Higuchi Model R^2^ k_H_(h^−1/2^)	0.9481 1.777	0.9452 1.841
Korsmeyer – Peppas Model R^2^ K_kp_(h^-n^) n	0.9931 0.192 0.835	0.9962 0.208 0.828
Hixson- Crowell Model R^2^ kHC	0.9956 0.000	0.9922 0.000
Hopfenberg Model R^2^ Khb n	0.9975 0.000 1.934	0.9963 0.000 1.700
Baker – Lonsdale Model R^2^ kBL	0.8404 0.000	0.8455 0.000
Makoid- Banakar Model R^2^ k_MB_ n k	0.9982 0.034 1.153 0.000	0.9966 0.126 0.921 0.000
Weibull Model R^2^ α β Ti	0.9985 7636.136 1.298 −26.001	0.9967 32508.582 1.500 −95.913
Peppas- Sahlin Model R^2^ k1 k2 m	0.9955 −2.272 0.845 0.334	0.9962 −0.078 0.229 0.409

All values are expressed as mean ± SD (*n* = 3).

### 3.7 *Ex vivo* permeation study

**Fig. 6:**
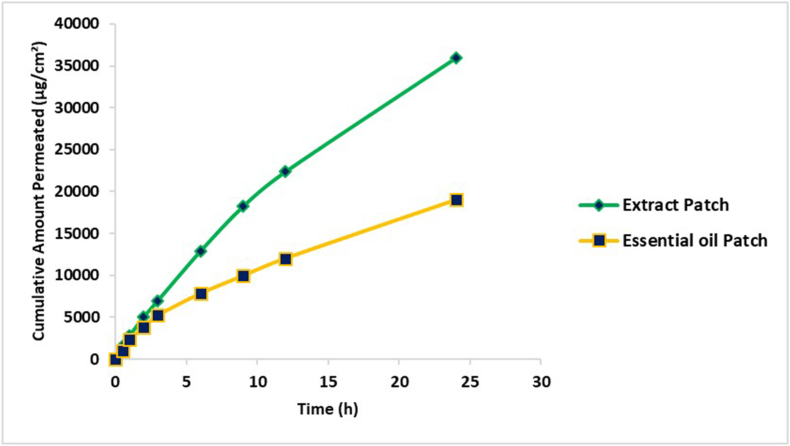
Ex vivo skin permeation profile drug from optimized patches. The extract patch indicates Ustukhuddūs hydro-alcoholic extract patch; the essential oil patch represents Ustukhuddūs essential oil patch. All values are expressed as mean ± SD (n = 3).

The *ex vivo* permeation profile of the drug serves as a vital tool for mitigating potential adverse drug effects. It aids in establishing a correlation with the *in vivo* pharmacokinetic behaviour of the drug. In this study, the cumulative amounts of drug permeated, measured in micrograms per square centimeter (μg/cm^2^), were calculated and plotted against time (Fig. 6). The *ex vivo* skin permeation study of UHAE and UEO patches was carried out in a modified Franz diffusion cell using newborn pig skin as a partition membrane structurally similar to human skin. The mean cumulative amount of drug permeated from developed UHAE and UEO patches after 24 h was recorded to be 35,956 ± 0.24 and 19,001 ± 1.46 μg/cm^2,^ respectively. The cumulative permeation of UHAE and UEO patches was calculated to be 66.82 ± 1.74 and 76.41 ± 1.22 %, respectively. The percentage cumulative permeation of the developed UEO patch was significantly higher than that of the developed UHAE patch. Initially, there is a swift dissolution of the hydrophilic polymers when the film comes into contact with hydrated skin. This rapid dissolution leads to a significant accumulation of the drug on the skin surface, ensuring a consistent saturation of the skin with drug molecules. The presence of a permeation enhancer further amplifies the drug release from both hydrophilic and hydrophobic polymers. The faster drug permeation in the initial phase is because of the hydrophilic polymer HPMC. In contrast, the sustained drug release in the later phase is due to the hydrophobic co-polymer EC acting as a release-controlling polymer. The sustained effect of the developed UHAE and UEO patches demonstrates their ability to consistently maintain therapeutic levels over an extended duration, aligning with the study's objective of achieving drug release for up to 24 h. While a substantial level of sustained release was accomplished, it's important to note that complete drug release at the 24-h mark was not achieved.

### 3.8 Skin penetration study

The result of the Skin penetration study found that the residual amount of UHAE and UEO accumulated in the stratum corneum layer of pig skin was 26.06 ± 1.81 and 18.96 ± 1.35 %, respectively. It indicates that the application of developed patches may prove beneficial for a period longer than 24 h, resulting in a reduction of consequences due to frequent dosing.

## 4 Conclusion and limitations

In this study, Unani transdermal patches incorporating *Ustukhuddūs* extract and essential oil marked a significant evolution in Unani dosage forms, specifically the transformation from *Ḍimād* (Paste). The developed patches demonstrated favorable physico-chemical compatibility, non-irritating properties, and physical stability, along with optimal sustained release characteristics, ensuring their safety profile for human use. Moreover, the transdermal patch containing *Ustukhuddūs* essential oil surpassed its counterpart with *Ustukhuddūs* extract in terms of physicochemical profile, drug release, and permeation. Thus, this study is a first of its kind to report that *Ustukhuddūs* can be effectively formulated into transdermal patches, offering advantages over traditional dosage forms and maintaining sustained release characteristics. While these findings are promising, further investigations are imperative to validate the outcomes and assess the potential therapeutic efficacy of the developed *Ustukhuddūs* patches in treating dementia and other neurodegenerative disorders through rigorous pre-clinical and clinical studies. This study concludes with a call for continued exploration of the applications and benefits of *Ustukhuddūs* in transdermal delivery systems.

## Ethical statement

Ethical clearance was obtained from the Institutional Animal Ethics Committee (IAEC) vide No. IAEC/6/19/IA/07, National Institute of Unani Medicine, Bengaluru, INDIA.

## Data availability statement

All the data included in article/supplementary material/referenced in article.

## CRediT authorship contribution statement

**Farhath Fathima A:** Writing – original draft, Visualization, Methodology, Investigation. **Imran Khan:** Writing – original draft, Validation, Software, Investigation, Formal analysis. **Mohammed Irfhan N:** Writing – review & editing, Software, Formal analysis, Data curation. **Zaheer Ahmed N:** Writing – review & editing, Methodology, Investigation. **Noman Anwar:** Writing – original draft, Methodology, Formal analysis, Data curation. **Mohd Shahnawaz Khan:** Writing – review & editing, Funding acquisition, Formal analysis. **Dharmendra Kumar Yadav:** Writing – review & editing, Investigation, Funding acquisition, Formal analysis. **Shariq Shamsi:** Writing – original draft, Visualization, Validation, Software, Resources, Methodology, Investigation, Formal analysis, Conceptualization. **Anas Shamsi:** Writing – original draft, Supervision, Methodology, Formal analysis, Data curation, Conceptualization.

## Declaration of competing interest

The authors declare that they have no known competing financial interests or personal relationships that could have appeared to influence the work reported in this paper.
